# Contrast-enhanced spectral mammography in recalls from the Dutch breast cancer screening program: validation of results in a large multireader, multicase study

**DOI:** 10.1007/s00330-016-4336-0

**Published:** 2016-04-20

**Authors:** U. C. Lalji, I. P. L. Houben, R. Prevos, S. Gommers, M. van Goethem, S. Vanwetswinkel, R. Pijnappel, R. Steeman, C. Frotscher, W. Mok, P. Nelemans, M. L. Smidt, R. G. Beets-Tan, J. E. Wildberger, M. B. I. Lobbes

**Affiliations:** 1 Departments of Radiology and Nuclear Medicine, Maastricht University Medical Center, P.O. Box 5800, 6202 AZ Maastricht, The Netherlands; 2 Department of Radiology, University Hospital Antwerp, Edegem, Meirstraat 61, 2890 St., Amands, Belgium; 3 Department of Radiology, University Medical Center Utrecht, Heidelberglaan 100, 3584 CX Utrecht, The Netherlands; 4 Department of Epidemiology, Maastricht University, P.O. Box 616, 6200 MD Maastricht, The Netherlands; 5 Department of Surgery, Maastricht University Medical Center, P.O. Box 5800, 6202 AZ, Maastricht, The Netherlands; 6 GROW School for Oncology and Developmental Biology, Maastricht University Medical Centre, P.O. Box 616, 6200 Maastricht, The Netherlands

**Keywords:** Breast cancer, Contrast-enhanced spectral mammography, CESM, Contrast-enhanced dual energy mammography, CEDM

## Abstract

**Objectives:**

Contrast-enhanced spectral mammography (CESM) is a promising problem-solving tool in women referred from a breast cancer screening program. We aimed to study the validity of preliminary results of CESM using a larger panel of radiologists with different levels of CESM experience.

**Methods:**

All women referred from the Dutch breast cancer screening program were eligible for CESM. 199 consecutive cases were viewed by ten radiologists. Four had extensive CESM experience, three had no CESM experience but were experienced breast radiologists, and three were residents. All readers provided a BI-RADS score for the low-energy CESM images first, after which the score could be adjusted when viewing the entire CESM exam. BI-RADS 1-3 were considered benign and BI-RADS 4-5 malignant. With this cutoff, we calculated sensitivity, specificity and area under the ROC curve.

**Results:**

CESM increased diagnostic accuracy in all readers. The performance for all readers using CESM was: sensitivity 96.9 % (+3.9 %), specificity 69.7 % (+33.8 %) and area under the ROC curve 0.833 (+0.188).

**Conclusion:**

CESM is superior to conventional mammography, with excellent problem-solving capabilities in women referred from the breast cancer screening program. Previous results were confirmed even in a larger panel of readers with varying CESM experience.

***Key Points*:**

• *CESM is consistently superior to conventional mammography*

• *CESM increases diagnostic accuracy regardless of a reader*’*s experience*

• *CESM is an excellent problem*-*solving tool in recalls from screening programs*

## Introduction

In breast imaging, mammography plays a pivotal role in breast cancer detection and evaluation. Although the diagnostic accuracy of conventional mammography has improved significantly during the last decade due to the introduction of full-field digital mammography (FFDM), its accuracy remains dependent on the density of the fibroglandular tissue [1].

Several new mammographic techniques have been introduced to improve FFDM’s diagnostic accuracy, the most recent one being contrast-enhanced spectral mammography (CESM). Previous studies have shown that CESM is superior to FFDM for breast cancer detection, even equalling the performance of breast MRI [2–4]. It was also demonstrated that CESM was clinically feasible even in a study population with a low disease prevalence, i.e., recalls from a breast cancer screening program [5]. Although all diagnostic performance parameters in this study improved when using CESM, the most important changes were observed regarding specificity (increasing from 42 % to 87.7 %) and positive predictive value (PPV, increasing from 39.7 % to 76.2 %). These results showed that in this population CESM has great potential as a problem-solving tool. However, they were based on an interim analysis of the institution’s first 113 patients, and readings were performed by only two radiologists who were experienced in reading CESM exams.

Reproducibility of initial findings is an important step in the evaluation of every new diagnostic technique. Therefore, we aimed to compare the diagnostic performance of FFDM and CESM using a larger panel of radiologists with different levels of CESM experience [5].

## Materials and methods

### Patient selection

For this retrospective study, the requirement for obtaining informed consent was waived by the local ethics committee. All women recalled from the breast cancer screening program who were referred to our institution for assessment in the period from November 2012 until October 2013 were eligible to undergo CESM. Women with a known allergy for iodinated contrast agents and those who had an increased risk for developing contrast induced nephropathy were excluded. The latter was established using the ESUR guidelines on Contrast Media, as stated by the European Society of Urogenital Radiology (ESUR) [6].

### Imaging protocol and analysis

The principle of the CESM technique was described elsewhere [7]. In short, a low-energy (LE) and a high-energy image (HE) are obtained of both breasts in the standard mediolateral oblique (MLO) and craniocaudal (CC) views. The LE image provides maximal soft tissue contrast and is similar to a conventional mammogram [8–10]. The HE image is not of diagnostic quality and is used for post-processing purposes only. Both images are used to create a recombined image which shows enhancement of lesions [7].

All CESM exams were performed on a single CESM unit (Senographe* Essential with Senobright* upgrade, GE Healthcare, Chalfont St Giles, United Kingdom) using a non-ionic, monomeric, low-osmolar contrast agent at a dose of 1.5 mL/kg body weight (iopromide, Ultravist® 300, Bayer Healthcare, Berlin, Germany). Iodinated contrast was intravenously administered with a flow rate of 3 mL/s two minutes prior to image acquisition. Both breasts were imaged in MLO and CC views with additional views to be requested by the radiologist if deemed necessary at the time of the exam. Patients were monitored for a minimal period of 30 minutes afterwards to rule out late adverse contrast reactions.

The panel of readers consisted of seven dedicated breast radiologists and three residents. Of the dedicated breast radiologists, four had 2 years of experience with CESM (the experienced CESM users). Their range of experience with conventional mammography was 2 to 6 years. The remaining three dedicated breast radiologists had between 3 and 25 years of experience with mammography but no previous experience whatsoever regarding CESM. The residents had limited CESM and mammography experience (8 weeks full time as part of their residency) and were trained in that period by the experienced CESM radiologists.

The panel of readers was allowed to learn the reason of referral from the screening program (similar to everyday clinical practice), and started by evaluating the LE image first. An initial breast imaging reporting and data system (BI-RADS) score of 1 to 5 had to be provided before evaluating the entire CESM exam, including both the LE and recombined images, during the same reading session. The reader was then allowed to upgrade or downgrade their BI-RADS score if deemed necessary. All radiologists were blinded for each other’s scores, previous or follow-up examinations and final diagnosis. Readers were divided into three subgroups: experienced CESM readers, non-experienced CESM readers and resident readers.

### Standard reference procedures used to assess true disease status

To assess the true disease status of a recalled patient, one of the strategies below was followed [11].

In the case of suspicious calcifications or masses, a biopsy was performed under ultrasound guidance or stereotactic guidance with histology serving as gold standard. In all cases of cysts, a targeted ultrasound examination was performed in combination with aspiration of the cyst to prove its non-solid nature. In cases where superposition of normal fibroglandular tissue was suspected, at least one additional view of the breast containing the suspicious lesion followed by targeted ultrasound was performed. If no abnormality was found on additional imaging, women were discharged according to the NHS Breast Screening Program (NHSBSP) Clinical Guidelines for Breast Cancer Screening Assessment and our national guidelines [12, 13].

### Statistical analysis

BI-RADS scores 1–3 were considered benign and BI-RADS 4–5 malignant. Using these cutoff values, sensitivity, specificity, positive predictive value (PPV) and negative predictive value (NPV) of the readers were calculated. Interpretation of images from the same set of patients by multiple readers are likely to be correlated. Moreover, because FFDM and CESM were performed in the same study population, the results of both tests are also correlated. Ignoring correlation can lead to misleadingly small estimates of the standard error and consequently to 95 % confidence intervals which are too small. To adjust for the correlated data structure, variance of the sensitivity and specificity for all readers and for subgroups of readers (experienced in CESM reading, non-experienced in CESM reading and residents) were adjusted with the variance inflation factor (VIF). The variance of the difference in sensitivity and specificity between FFDM and CESM was estimated based on these adjusted variances and the covariance between FFDM and CESM results. Adjusted 95 % confidence intervals (CI) were derived using an excel spreadsheet provided by Genders et al. [14]. Receiver operating characteristic (ROC) curves were constructed for both imaging modalities and areas under the curve (AUC), with corresponding 95 % CI calculated using bootstrap analyses (2000 repetitions). The DeLong test was used for paired comparison of the AUC of FFDM and CESM [15]. Fleiss’ generalized kappa coefficient was used to determine the inter-rater reliability for the image analysis of both FFDM and CESM.

In a separate analysis, false negative and false positive findings were further evaluated. All false negatives were analyzed to identify potential CESM pitfalls. For false positives findings, cases were included for this subanalysis if five or more readers (i.e., more than half of the reading panel) scored this case as being false positive on CESM. All statistical analyses were performed using SPSS (IBM Corp. Released 2011. IBM SPSS Statistics for Windows, Version 20.0. Armonk, NY: IBM Corp.) and the pROC package in R (Version: 1.7.2 released on 6 April 2014) [15]. *P* values ≤ 0.05 were considered statistically significant.

## Results

A total of 199 consecutive patients who were referred to our institution from the breast cancer screening program underwent CESM as part of their workup (mean age 58.4 years, range 49–75 years). Most recalls were caused by masses (76.4 %), followed by calcifications (15.1 %), asymmetry (5.0 %) and architectural distortions (3.5 %). At final diagnosis, 29.6 % of the cases proved to be malignant. Detailed patient characteristics were summarized in Table 1.

**Table 1:** Patient characteristics: age, final diagnosis for malignant and benign lesions and subtypes of invasive cancers given

Age	Years
Mean	58.4
Standard deviation	8.1
Range	49–75
Final Diagnosis	Percentage (*n* = 199)
Malignant	
Invasive ductal carcinoma	22.1
Invasive lobular carcinoma	4.0
Ductal carcinoma in situ	2.5
Invasive mucinous carcinoma	0.5
Invasive micropapillary carcinoma	0.5
Benign	
Fibroadenoma	5.5
Simple cyst	19.1
Reactive changes / benign	1.5
Apocrine changes / metaplasia	3.5
Papilloma	1.5
Superposition	28.1
Cillindrical cell changes	2.0
Old hematoma	0.5
Inflammation	0.5
Intramammary lymphnode	2.0
Sclerosing adenosis	1.0
Atypical lobular hyperplasia	0.5
Ductectasia	1.0
Fibrosis	1.5
Ductal hyperplasia	1.0
Lobular carcinoma in situ	0.5
Flat epithelial atypia	0.5
Total	100.0
Invasive breast cancer subtypes	Percentage of all invasive cancers
ER positive	92.3
PR positive	80.8
HER2/neu positive	12.2
Grade 1	27.3
Grade 2	47.3
Grade 3	25.5

ER: estrogen receptor, PR: progesterone receptor, HER2/neu: Human Epidermal growth factor Receptor 2

**Fig. 1:**
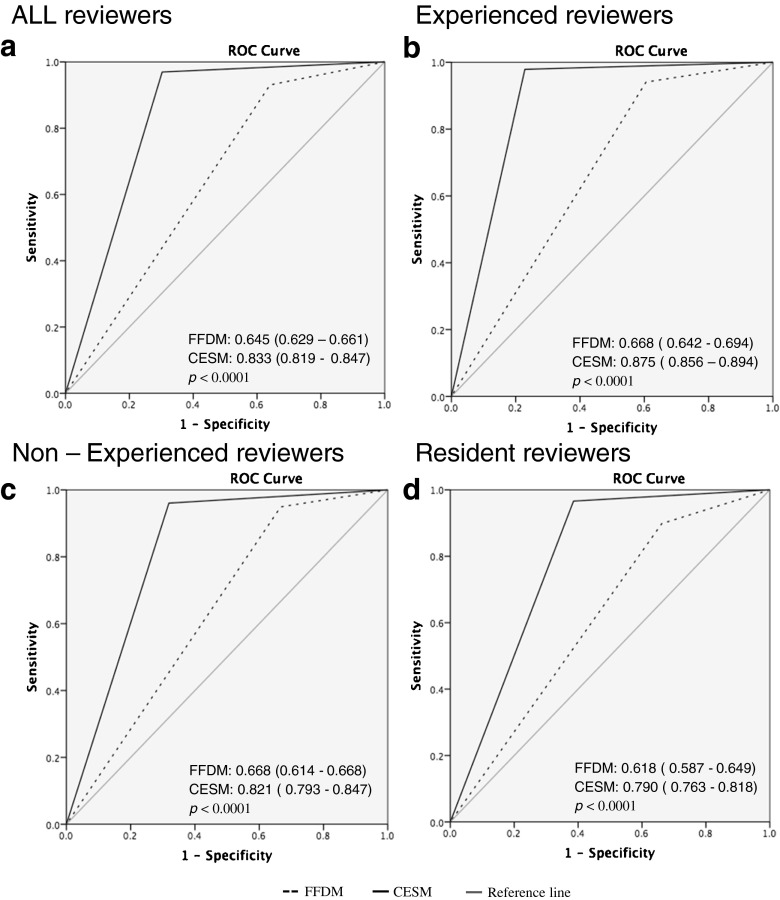
Average ROC curves for all readers (**a**), experienced CESM readers (**b**), experienced FFDM readers (**c**) and resident readers (**d**). AUC values for FFDM and CESM given with confidence intervals in parenthesis. Differences in AUC between FFDM and CESM were significantly increased for all subgroup of reader panels, *p*-values given per subgroup of reader panel

Table 2 presents detailed information of sensitivity and specificity for all readers. For the entire reading panel, diagnostic performance parameters improved when using CESM. Mean sensitivity increased from 93.0 % to 96.9 % and mean specificity from 35.9 % to 69.7 %. Mean PPV and NPV increased from 38.7 % and 92.6 % to 58.2 % and 98.2 %, respectively. The ROC curves showed an improvement in diagnostic performance for all readers when using CESM (Fig. 1). For all readers combined, the AUC value increased from 0.645 to 0.833 (*p* < 0.0001). Detailed results for the comparison of CESM and FFDM are presented in Table 3. Sensitivity increased for all reader panels, but was only significantly increased for resident readers (*p* = 0.011) and for all readers together (*p* = 0.0002). Looking at the difference in sensitivity and specificity for CESM and FFDM, both increased for all readers using CESM. For all subgroups of reader panels, specificity was significantly increased. The inter-rater variability was considered to be excellent with a kappa-value of 0.89.

**Table 2:** Diagnostic performance of FFDM and CESM for all ten readers. Diagnostic performance parameters were presented as percentages with 95 % confidence intervals in parentheses

Reader	Exam	Sensitivity	Specificity
Experienced CESM Reader 1	*FFDM*	86.4 % (75.0–93.9 %)	67.1 % (58.7–74.8 %)
	*CESM*	93.2 % (83.5–98.1 %)	86.4 % (79.6–91.6 %)
Experienced CESM Reader 2	*FFDM*	96.6 % (88.2–99.4 %)	26.4 % (19.3–34.5 %)
	*CESM*	98.3 % (90.8–99.7 %)	70.7 % (62.4–78.1 %)
Experienced CESM Reader 3	*FFDM*	94.9 % (85.8–98.9 %)	49.3 % (40.7–57.8 %)
	*CESM*	100.0 % (93.8–100.0 %)	75.7 % (67.7–82.6 %)
Experienced CESM Reader 4	*FFDM*	98.3 % (90.8–99.7 %)	15.0 % (9.5–22.0 %)
	*CESM*	100.0 % (93.8 %–100.0 %)	75.7 % (67.7–82.5 %)
	Mean FFDM	94.1 % (89.6–98.5 %)	39.5 % (19.7–59.2 %)
	Mean CESM	97.6 % (95.1–100 %)	77.1 % (71.5–82.7 %)
Non-Experienced CESM Reader 1	*FFDM*	98.3 % (90.8–99.7 %)	37.8 % (29.8–56.4 %)
	*CESM*	100.0 % (93.8–100.0 %)	67.1 % (58.7–74.8 %)
Non-Experienced CESM Reader 2	*FFDM*	96.6 % (88.2–99.5 %)	21.4 % (14.9–29.1 %)
	*CESM*	94.9 % (85.8–98.8 %)	64.3 % (55.7–72.2 %)
Non-Experienced CESM Reader 3	*FFDM*	89.9 % (79.1–96.1 %)	40.7 % (32.5–49.3 %)
	*CESM*	93.2 % (83.5–98.1 %)	72.8 % (64.7–80.0 %)
	Mean FFDM	94.9 % (90.8–99.0 %)	33.3 % (23.7–42.9 %)
	Mean CESM	95.9 % (92.9–98.9 %)	68.0 % (64.1–72.1 %)
Resident Reader 1	*FFDM*	89.8 % (79.1–96.1 %)	32.8 % (25.1–41.3 %)
	*CESM*	96.6 % (88.2–99.4 %)	58.5 % (49.9–66.8 %
Resident Reader 2	*FFDM*	93.2 % (83.5–98.0 %)	36.4 % (36.4–44.9 %)
	*CESM*	96.6 % (88.2–99.4 %)	64.2 % (55.7–72.2 %)
Resident Reader 3	*FFDM*	86.4 % (75.0–93.9 %)	32.1 % (24.5–40.5 %)
	*CESM*	96.6 % (88.2–99.5 %)	61.4 % (52.8–69.5 %)
	Mean FFDM	89.8 % (79.2–96 %)	33.7 % (28.6–42.2 %)
	Mean CESM	96.6 % (95.4–98.2 %)	61.3 % (52.8–69.5 %)
All Readers Mean	*FFDM*	93.0 % (90.3–95.8 %)	35.9 % (27.3–44.5 %)
	*CESM*	96.9 % (93.2–100.0 %)	69.7 % (64.8–74.6 %)

**Table 3:** Difference (∆) in sensitivity and specificity of CESM and FFDM with 95 % confidence intervals (CI) in parenthesis. *p* values < 0.05 are considered significant

	∆ Sensitivity CESM-FFDM (95 % CI)	*p* value (<0.05 significant)	∆ Specificity CESM-FFDM (95 % CI)	*p* value (<0.05 significant)
Experienced CESM readers	0.035 (-0.009–0.079)	*p* = 0.114	0.376 (0.193–0.559)	*p* = 0.000056
Non-experienced CESM readers	0.010 (0.036–0.056)	*p* = 0.667	0.348 (0.254–0.442)	*p* < 0.00001
Resident Readers	0.068 (0.016–0.120)	*p* = 0.011	0.276 (0.223–0.329)	*p* < 0.00001
All readers	0.038 (0.018–0.058)	*p* = 0.0002	0.338 (0.267–0.409)	*p* < 0.00001

CESM false negative findings

Ten cases (5 %) with false negative CESM findings were observed. An overview of the final diagnosis, tumour characteristics and the number of readers that scored the individual case as false negative is presented in Table 4. Three readers had no false negative scores with CESM. All other readers had at least one false-negative finding on CESM (median two cases, range 1–4).

**Table 4:** Diagnosis of false negative cases and the number of readers that scored them as false negative. Experience level is indicated of the number of readers that missed the lesion on CESM. In addition, lesion characteristics such as diameter (given in millimetres), histologic grade, DCIS grade and hormonal receptor status (ER, PR, HER2NEU) are given. Hormone receptor status in case of pure DCIS is not evaluated and therefore not available (n/a) for these cases

Final diagnosis	Number of readers scoring false negative				Tumour Characteristics				
Histology	Experienced CESM	Non- experienced CESM	Resident	Total	Diameter in mm	Grade	ER	PR	HER2/neu
Invasive ductal carcinoma	1	2	2	5	14	2	+	+	-
Invasive ductal carcinoma	1	1	1	3	22	2	+	+	+
Invasive mucinous carcinoma	-	2	-	2	18	1	+	+	-
Invasive ductal carcinoma	-	1	-	1	20	2	+	-	-
Invasive ductal carcinoma	1	-	-	1	7	1	+	+	-
Invasive ductal carcinoma	1	-	-	1	4	1	+	-	-
Invasive ductal carcinoma	-	-	1	1	16	2	+	+	-
Ductal carcinoma in situ	-	1	-	1	10	2-3	n/a	n/a	n/a
Invasive lobular carcinoma	-	-	1	1	5	2	+	+	-
Ductal carcinoma in situ	-	-	1	1	26	3	n/a	n/a	n/a

**Fig. 2:**
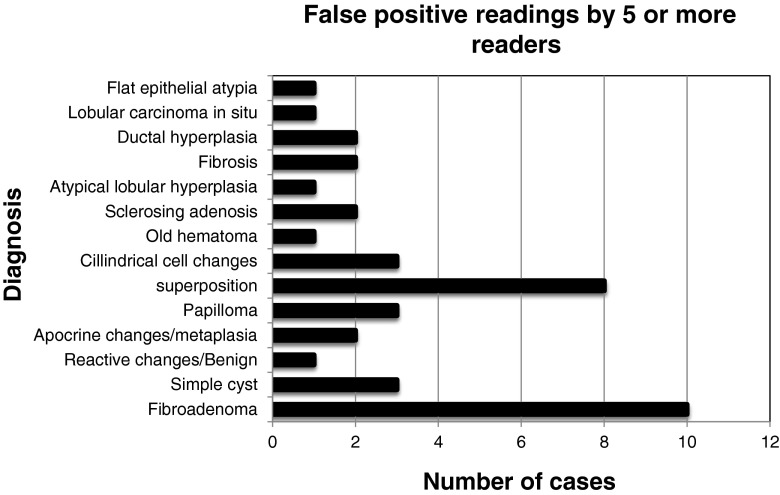
Overview of number of cases and diagnosis of false-positive findings. These cases were scored as false-positive cases by five or more readers

The mean number of false positive cases was 42 (21.1 %, range 19-58), with an average for the experienced CESM readers of 31 cases (15.6 %). The experienced FFDM readers showed an average of 37 false positive cases (18.6 %), whereas the residents showed 54 false positive findings (27.1 %). A total of 40 cases (20.1 %) were scored as false positive by five or more readers using CESM. In this subgroup, the most common causes were fibroadenomas (*n* = 10), followed by superposition densities (*n* = 8), and cysts (*n* = 3). A detailed summary of this subanalysis is presented in Fig. 2.

## Discussion

CESM is a promising new breast imaging modality. In CESM, an iodine-based contrast agent is intravenously administered, after which dual-energy mammography is performed. As a result, the radiologist can view a low-energy image (which is similar to a conventional FFDM) and a recombined image, showing areas of enhancement [7, 16]. In a previous study, it was shown that CESM is an excellent problem-solving tool for women recalled from the breast cancer screening program [5]. However, these results were based on an interim analysis of the institution’s first 113 cases read by only two radiologists experienced in CESM. Our current study shows that these results were reproducible, even in a large number of cases read by a panel of ten different radiologists with varying experience in reading CESM exams. Mean sensitivity increased from 93.0 % to 96.9 % and mean specificity from 35.9 % to 69.7 %. Mean PPV and NPV increased from 38.7 % and 92.6 % to 58.2 % and 98.2 %, respectively.

Several publications have studied the diagnostic performance of CESM (Table 5) [2–5, 8, 17–22]. In these studies, the mean sensitivity of CESM varied from 77.8 to 100.0 %, whereas mean specificity (if available) varied from 41 to 87.7 %. In some studies, specificity could not be calculated since all included subjects were diagnosed with breast cancer [3, 8, 19]. One study did not provide specificity, but accuracy instead [22].The disease prevalences in the other studies (except the study by Lobbes et al.) were higher than our population (range 36-100 %). The reported prevalence of 36 % concerned a study population where all included subjects had microcalcifications without an associated mass [21]. However, breast cancer prevalence in clinical practice is low. It is interesting to study the diagnostic performance of CESM in populations with low breast cancer prevalence since it should not result in a large number of false-positive findings.

**Table 5:** Studies comparing CESM and Mammography: number of patients included, sensitivity and specificity given for CESM

Study	Number of patients (*n*)	Sensitivity (%)	Specificity (%)	Disease prevalence (%)
Lewin et al. [17]	26	100	86.7	50
Dromain et al. [2]	144	93	63	56.3
Dromain et al. [18]	110	91.9	46	56.7
Fallenberg et al. [19]	80	100	-	100
Jochelson et al. [3]	52	96	-	100
Fallenberg et al. [8]^a^	118	94.7*/95**	-	100
Lobbes et al. [5]	113	100	87.7	28
Luczyńska et al. [4]	152	100	41	76
Cheung et al. [20]	89	92.7	67.9	72
Luczyńska et al. [22]	118	100	Not provided	68.6
Cheung et al. [21]^b^	52	90.9	83.78	37.7

Disease prevalence based on number of lesions analysed in the included population is given, calculated from data given in study

Annotations: ^a^Sensitivity for CESM alone (*) and for CESM in combination with mammography (**). ^b^Patients with microcalcifications only

For this reason, Lobbes et al. studied CESM’s diagnostic performance in women recalled from breast cancer screening, who had a breast cancer prevalence of 28.3 % [5]. They found that (when compared to FFDM) sensitivity increased from 96.9 % to 100.0 % and NPV increased from 97.1 % to 100.0 %. Interestingly, the largest improvements were observed for specificity and PPV, increasing from 42.0 % and 39.7 % to 87.7 % and 76.2 %, respectively. It was concluded that CESM was an excellent problem-solving imaging modality for recalls from the breast cancer screening program, able to detect breast cancer accurately, while establishing false-positive recalls confidently.

An important limitation of the study by Lobbes et al. was that two readers experienced with CESM read the cases. However, in order to become clinically implemented, the reproducibility of test results of every new diagnostic imaging modality should be validated, preferably in larger study populations using multiple readers. Therefore, we used a panel of ten different readers with different experience in FFDM and CESM to evaluate 199 consecutive CESM exams of women recalled from the breast cancer screening program. Our current results confirmed prior observations, with an increase of all diagnostic performance parameters when using CESM, especially with respect to specificity and PPV. These improvements were observed for all readers, independent of their level of CESM experience. Current results are in line with another previously published study with relatively lower disease prevalence. Luczyńska et al. studied 157 breast lesions (breast cancer prevalence 38.3 %) using both FFDM and CESM [4]. Sensitivity of CESM was 100 %, with a PPV and NPV of 77 % and 100 %, respectively, with an AUC of 0.86. However, in their study only a single reader was used to view the exams.

**Fig. 3:**
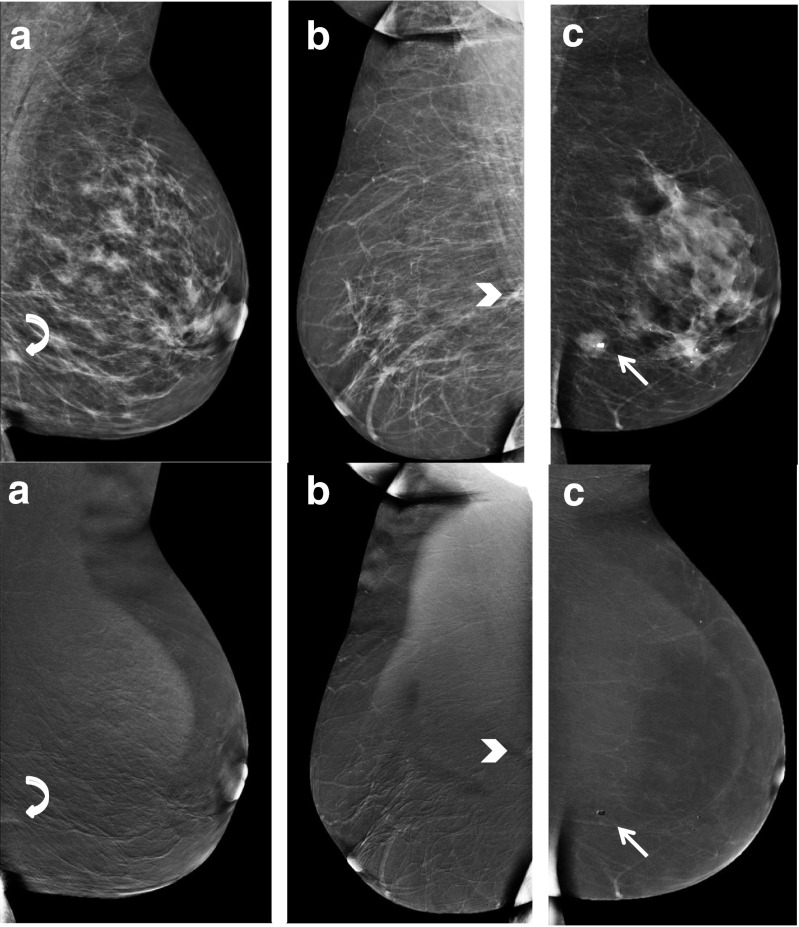
Example of false negatives cases. Low-energy images at the top with corresponding re-combined images underneath. A: infiltrating grade 2 ductal carcinoma with grade 3 ductal carcinoma in situ (*curved arrow*), B: invasive grade 2 ductal carcinoma (*arrow head*) and C: grade 1 mucinous carcinoma (*straight arrow*)

CESM has potential pitfalls, resulting in both false negative or false positive findings. In a study by Thibault et al., six false negative findings were observed: two invasive ductal carcinomas outside the field of view and four invasive lobular cancers [23]. Fallenberg et al. (using three readers) found that when CESM was solely used, one cancer was missed by all readers, four cancers were missed by two, and three by one reader [8]. In our study, a total of ten false negative cases were observed, scored incorrectly by one or more readers. The cancers that were overlooked by more than one reader were analyzed case-by-case. These were: one invasive grade 2 ductal carcinoma missed by five readers, one invasive grade 2 ductal carcinoma missed by three readers, and one grade 1 invasive mucinous carcinoma missed by two readers. These cases are illustrated in Fig. 3. Two cases consisted of a focal asymmetry with ill-defined margins, only partly visible on the MLO view only. These lesions showed only subtle or no enhancement on the recombined images (Fig. 3a and b). Among the readers that missed these lesions were experienced CESM users as well as non-experienced CESM users and residents. Future developments such as computer aided detection systems for CESM or contrast-enhanced digital breast tomosynthesis could potentially reduce the risk of missing these types of lesions. The third case (missed by two readers) consisted of an ill-defined mass visible on both CC and MLO views with a central coarse calcification without any enhancement on the recombined images. Despite the lack of enhancement, it does not represent a typical ‘eclipse’ sign, which is the CESM appearance of a cyst, consisting of a dark ‘void’ on the recombined images combined with a subtle rim enhancement, resembling a solar eclipse [5]. This atypical appearance of the eclipse sign together with its irregular margin warranted additional targeted ultrasound. Final pathology showed a grade 1 mucinous carcinoma (Fig. 3c). Mucinous carcinomas can be a CESM pitfall due to lack of enhancement. This case demonstrates that readers should not only focus on the recombined images. They are an adjunct to the mammographic images, not a replacement.

CESM also generates false positive findings. In the study by Badr et al. enhancement was observed in 33 % of 27 benign lesions [24]. Jochelson et al. detected two false positive results in 52 patients (4 %) using CESM [3]. Lobbes et al. detected five false positive findings in a population of 113 women [5]. Luczyńska et al. found 35 (20 %) false positive lesions with CESM compared to 50 (29 %) with conventional mammography [4]. Similar to our observations, most of these lesions were caused by fibroadenomas (*n* = 26) or some other benign solid breast lesion. Although these findings resulted in tissue sampling that could have been avoided, its prevalence is low and does not outweigh the improved cancer detection rates caused by CESM when compared to FFDM.

This study showed that CESM remains an excellent problem-solving tool for patients recalled from breast cancer screening, even when radiologists less experienced in CESM are reviewing the images. This implies that reading CESM exams has hardly any learning curve. Introduction into everyday clinical practice is safe and feasible [5]. Using CESM in recalled patients increases specificity and PPV, thus providing the radiologist with a confident final diagnosis in cases of false positive recalls. For example, if recalled patients have a negative CESM exam, the high NPV rules out the presence of breast cancer, preventing people from undergoing additional exams (such as breast MRI) or follow-up exams. Nevertheless, prospective randomized controlled trials are necessary to compare the standard work-up using conventional breast imaging with CESM-based work-up, in order to accept CESM as a primary imaging tool in the work-up of recalled patients.

### Study limitations

Our study had some limitations. Earlier results were previously published and consisted of 113 cases read by two experienced CESM viewers [5]. In the current study, these two readers were again participating in the scoring of the exams, thus re-scoring these exams. However, the data used was anonymized and the time period between the two scoring rounds was more than one year, minimizing the chances of introducing recall bias in these 113 cases. The remaining 86 cases were also new to these two readers. To prove that no recall bias was introduced, we performed additional analyses. In the previous publication, the AUC of ROC curve was 0.779 for mammography, increasing to 0.976 using CESM [5]. These were the results of 113 cases. After more than one year, two readers reviewed these 113 cases again as part of the current study, achieving a diagnostic performance as expressed by the AUC value of 0.831 for mammography and 0.971 using CESM. The AUC value of the final 86 (completely new) cases was 0.881 for mammography and 0.977 for CESM. This shows that no recall bias was introduced during the re-reading of the first 113 cases by these two readers. For the remaining eight readers, all 199 cases were completely new. For reasons of comparison, we decided to include the complete data of the two experienced CESM reviewers to provide an overview of the performance of each reader for the entire case collection. However, in order to further assess the reproducibility of these results, it would be valuable to conduct a study consisting of an entirely different population, preferably in different institutes using units of different vendors, which are now becoming commercially available. Second, there was no follow-up of cases with superposition of fibroglandular tissue as final diagnosis. However, our current imaging strategy of these cases complies with the NHSBSP’s Clinical Guidelines for Breast Cancer Screening Assessment [12]. This strategy is safe, with the chances of overlooking breast cancer being minimal, as was additionally proven by an institutional quality control covering almost 600 recalls from screening (personal communication). Third, all cases were recalls from the national breast cancer screening program. This introduced some selection bias since all patients were pre-selected by two screening radiologists. In addition, readers were not blinded for the reason of referral. However, the latter two limitations reflect everyday clinical practice of the work-up of recalled women. Finally, the additional value of (targeted) ultrasound next to FFDM was not taken into account since we wished to focus on the additional value of adding contrast and the recombined images to conventional mammography. Indeed, additional ultrasound could also clarify some findings that proved to be benign (such as a cyst). Since Dromain et al. showed that CESM is also superior to mammography and ultrasound combined [2], it would still be recommendable to use CESM as a primary imaging tool for recalled patients.

## Conclusion

The diagnostic performance of CESM is superior to FFDM in women recalled from the breast cancer screening program, confirming previously published results. Even when used by less experienced CESM readers, CESM increases all diagnostic accuracy parameters, especially specificity and positive predictive value.

## Abbreviations

95 % CI: 95 % confidence interval
AUC: Area under the curve
BI-RADS: Breast imaging reporting and data system
CC: Craniocaudal
CESM: Contrast-enhanced spectral mammography
ESUR: European Society of Urogenital Radiology
FFDM: Full-field digital mammography
LE: Low-energy
MLO: Mediolateral oblique
MRI: Magnetic Resonance Imaging
NHS: National Health Service
NPV: Negative predictive value
PPV: Positive predictive value
ROC: Receiver operating characteristic
TN: True negative
TP: True positive
VIF: Variation inflation factor
